# Efficient Gene Editing in Fish Primary Germline Stem Cells

**DOI:** 10.3390/ijms27146294

**Published:** 2026-07-15

**Authors:** Yuanyuan Zhan, Tingting Luo, Yuhua Sun

**Affiliations:** 1 Institute of Hydrobiology, Chinese Academy of Sciences, Wuhan 430072, China; zhanyuanyuan23@mails.ucas.ac.cn (Y.Z.);; 2 University of Chinese Academy of Sciences, Beijing 100049, China; 3 College of Life Sciences, Huzhou Normal University, Huzhou 313000, China

**Keywords:** *Monopterus albus*, germline stem cells, CRISPR/Cas9, microchannel cell transfection system

## Abstract

Genome editing by the CRISPR/Cas9 system is widely used for production of gene-modified animals, including fish. However, efficient gene editing in fish cultured cells, in particular germline stem cells (GSCs), is challenging, likely due to the difficulty in transfecting these cells. The ricefield eel (*Monopterus albus*), a sequential hermaphroditic species, is a freshwater fish of significant economic value in China. In this work, we report a simple method that can achieve high gene editing efficiency in primary GSCs of fish species, including ricefield eel. High transfection efficiency (50–95%) is achieved in fish GSCs by using a microchannel-based cell transfection system. Gene editing efficiency of up to 60% in primary ricefield eel GSCs is achieved using an integrated CRISPR/Cas9 vector strategy. High gene editing efficiency is also achieved in gibel carp (*Carassius gibelio*) GSCs and the medaka spermatogonial stem cell line SG3, demonstrating the general applicability of our method. Our data suggest that efficient transfection is key to high gene editing efficiency in fish cultured GSCs. This study establishes an efficient and reliable gene editing system for fish GSCs, which may facilitate the creation of new germplasm of genetically difficult-to-breed fish by combining GSC transplantation with gene editing techniques.

## 1. Introduction

The CRISPR/Cas9 system is widely used to generate genetically modified animals, including fish. Recently, a growing number of studies have focused on gene editing in fish cultured cells using various methods. For example, genome editing has been successfully achieved in zebrafish cultured cells with a CRISPR/Cas9 ribonucleoprotein complex [1]. In addition, CRISPR/Cas9-mediated gene editing has been reported in the CHSE-214 salmon cell line using a lentiviral delivery system [2,3,4,5,6,7,8]. However, compared to their mammalian counterparts, efficient gene editing in fish cultured cells remains challenging [9,10] for several reasons. First, transfection efficiency in fish cells is typically low [11]. Second, the current Cas9 proteins and CRISPR plasmid vectors used in the CRISPR/Cas9 system are not optimized for fish cells. Specifically, the membrane structure of fish cells exhibits lower fluidity and higher density at low temperatures, with a greater proportion of long-chain saturated fatty acids in their phospholipids. This distinctive property significantly hinders the transmembrane transport efficiency of exogenous DNA molecules [12]. Third, the selection of appropriate U6 promoters is shown to be critical for achieving efficient gene editing [13].

Germline stem cells (GSCs) are highly specialized, undifferentiated cells located within mature gonads [14,15]. Successful isolation and purification of GSCs have been reported in multiple fish species [16,17,18,19,20,21]. As an assisted reproductive technology, GSC transplantation enables the surrogate production of donor-derived gametes. This technique has gained increasing popularity in aquaculture [22,23,24,25] because it helps overcome reproductive barriers, shorten reproductive cycles, increase fecundity, and reduce broodstock management costs. Importantly, combining GSC transplantation with genome editing techniques offers a powerful approach to accelerate selective/targeted breeding, sex control breeding, and the creation of new germplasm [26,27]. Unfortunately, gene editing in fish GSCs remains highly challenging [1,3,11,28,29,30,31,32]. Given that efficient transfection is critical for achieving high gene editing efficiency, there is an urgent need to develop better transfection methods suitable for fish GSCs.

The ricefield eel, also known as the swamp eel, is an economically important freshwater fish species in China [33,34,35]. Renowned for its nutritional value and unique flavor, it enjoys strong consumer demand and holds significant potential for the aquaculture industry [36]. In 2025, the annual output of ricefield eel reached over 35 tons. Ricefield eel is a protogynous hermaphroditic fish, which begins life as a female and subsequently transitions into a male through an intersex stage [37]. This unique reproductive mode poses considerable challenges for genetic breeding and the creation of new germplasm using conventional methods [38]. Currently, there is no new breeds of this species. Combining gene editing with GSC transplantation techniques offers a promising solution to overcome these difficulties.

Aromatase (*cyp19a1*) is a key rate-limiting enzyme in the estrogen synthesis pathway, responsible for converting the enone ring in androgen precursors (testosterone and androstenedione) into a phenolic group, thereby producing estrogens (17β-estradiol and estrone). The gonadal *cyp19a1a* gene plays a crucial role in ovarian differentiation and maintenance in fish [39,40]. Foxl2, a member of the forkhead transcription factor superfamily, is a transcription factor specifically expressed in ovarian granulosa cells [41]. Studies have shown that *foxl2* can directly regulate *cyp19a1a* expression [42]. During the transition from ovary to testis in ricefield eel, the mRNA expression levels of *foxl2* and *cyp19a1a* gradually decrease [43,44,45], while the DNA methylation levels in their promoter regions significantly increase [46]. Performing gene editing on *foxl2* and *cyp19a1a* in GSCs followed by transplantation enables functional studies of these genes, which will be useful for developing strategies for genetic breeding and sex-control of ricefield eel in future aquaculture practice.

In this study, we evaluated the transfection efficiency of three different methods (chemical transfection reagent, electroporation, and microchannel-based cell transfection) in ricefield eel GSCs using a reporter vector. Our results demonstrated that microchannel-mediated transfection achieves high transfection efficiency while causing minimal cell death. Consequently, this approach enabled high-efficiency gene editing in primary isolated GSCs.

## 2. Results

### 2.1. Isolation and Purification of Monopterus albus Germ Stem Cells

**Figure 1:**
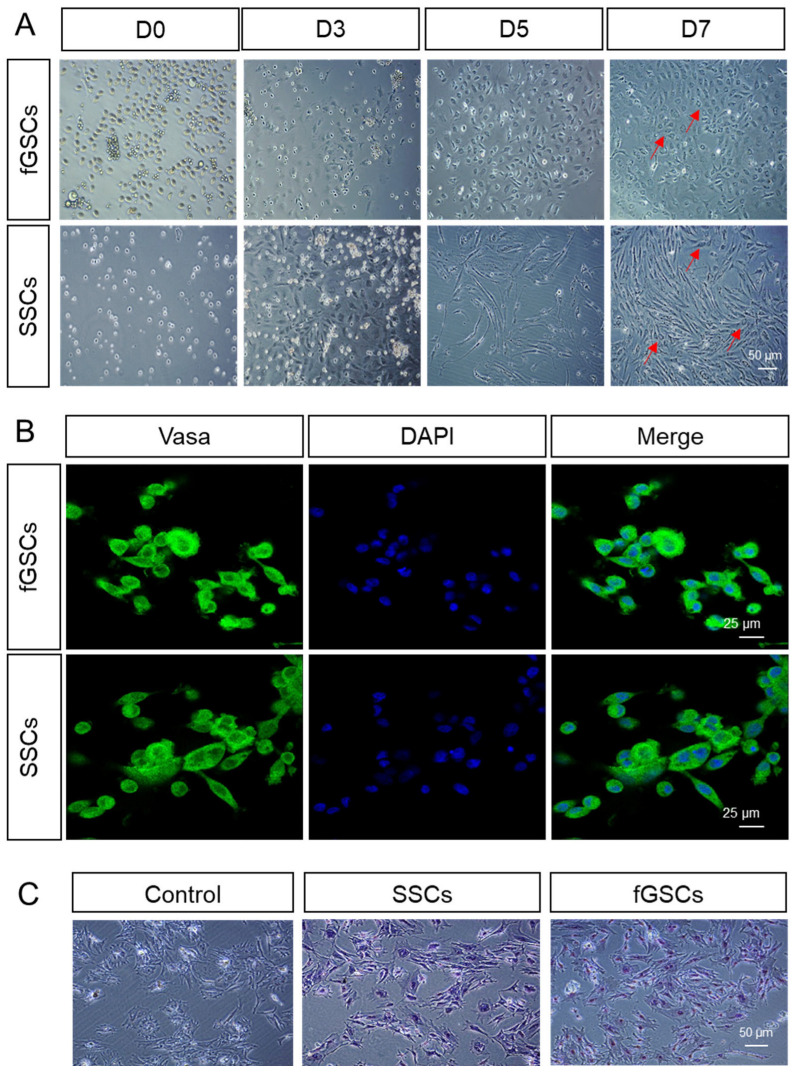
Isolation and purification of *Monopterus albus* GSCs. (**A**) Cell morphology of GSCs isolated from ovary and testis at the indicated time points. Red arrows pointing to the cells with cobblestone morphology. (**B**) Immunofluorescence staining results of Vasa in fGSCs and SSCs. (**C**) Representative AP staining images of fGSCs and SSCs.

Gonadal cells were successfully isolated from randomly selected 1.5-year-old female and 4-year-old male fish. By day 5 of culturing, the attached GSC-like cells from both female and male animals exhibited typical germline stem cell morphology, displaying a round or oval shape with a high nucleoplasmic ratio (mean nucleus-to-cytoplasm ratio: 0.72 ± 0.05, *n* = 50 cells), prominent nucleoli, and clear cell borders (Figure 1A). The average cell diameter was 12.5 ± 1.8 μm for fGSCs and 11.9 ± 1.5 μm for SSCs, which are consistent with previously reported GSC dimensions in teleosts [21].

To verify the stem cell identity of these cells, immunofluorescence (IF) staining experiments were performed using antibodies against Vasa, a germline stem cell marker (Figure 1B). The results showed that nearly all attached cells at day 7 were Vasa-positive (green fluorescence), compared to the negative control (Figure 1B). Alkaline phosphatase (AP) staining was performed (Figure 1C). Within the formed clonal colonies, the vast majority of cells exhibited strong AP activity (stained purplish-red). Thus, female GSCs (fGSCs) and spermatogonial stem cells (SSCs) can be isolated and purified, as evidenced by morphological features, alkaline phosphatase activities, and abundant expression of Vasa protein. We primarily used fGSCs from immature ovaries for subsequent experiments due to its relative abundance in the gonads [47].

### 2.2. Comparison of Transfection Efficiency of Different Methods in fGSC

**Figure 2:**
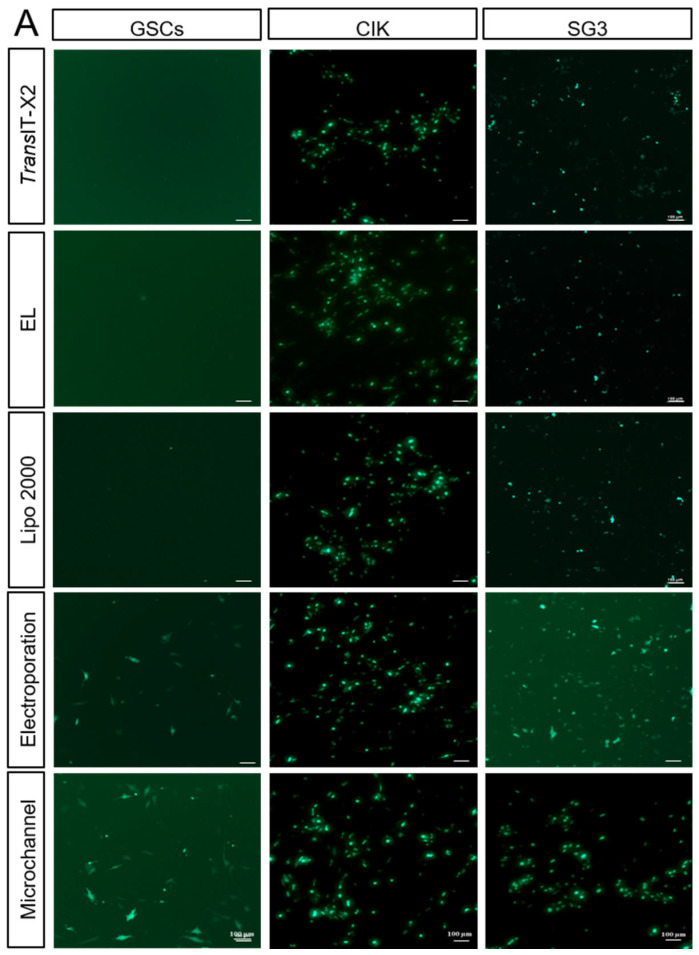

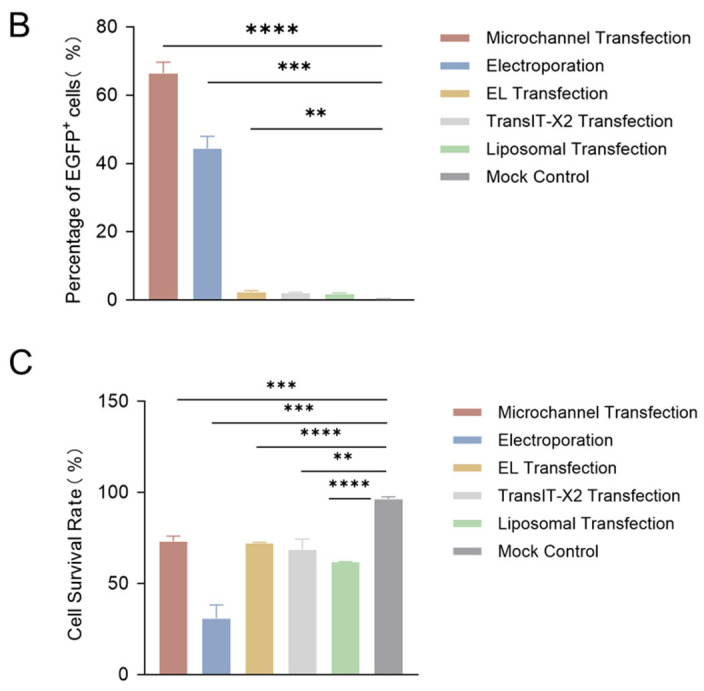
Comparison of transfection efficiency of different methods in fGSCs. (**A**) Three distinct cell types—fGSCs from swamp eel, CIK cells from grass carp (*Ctenopharyngodon idellus*), and the SG3 spermatogonial stem cell line from medaka—were transfected in 12-well plates using various chemical transfection reagents. Cells were monitored under a fluorescence microscope to assess EGFP fluorescence expression 24 h after transfection with the pCS2-EGFP plasmid. *Trans*IT-X2: transfection was performed using the *Trans*IT-X2 Dynamic Delivery System; EL: cells were transfected with the TransIntro EL Transfection Reagent; Lipo 2000: the Hieff Trans™ Liposomal 2000 Transfection Reagent was used; Electroporation: transfection was conducted using the Invitrogen™ Neon™ Transfection System; Microchannel: cells were transfected employing a microchannel-based transfection instrument (MDMP E-1). Scale bar, 100 µm. (**B**) Quantification of the percentage of EGFP-positive fGSCs in different groups. Data are shown as means ± SD from three independent biological replicates (*n* = 3 fish per group). For each biological replicate, three technical replicates were performed. The asterisks above the error bars indicate statistical significance (*p* < 0.05). (**C**) Quantification of the proportion of surviving fGSCs across different groups. Data are shown as means ± SD from three independent biological replicates (*n* = 3 fish per group). For each biological replicate, three technical replicates were performed. **: *p* < 0.01; ***: *p* < 0.001; ****: *p* < 0.0001.

A prerequisite for successful genome editing is achieving high transfection efficiency in the target cells. Ricefield eel fGSCs were first transfected using different chemical transfection reagents, including the Hieff Trans™ Liposomal 2000 Transfection Reagent, the TransIT-X2 Dynamic Delivery System, and the EL Transfection Reagent. The pCS2-eGFP plasmid was used as a reporter to monitor transfection efficiency. As expected, 2 days after transfection with each chemical reagent, abundant GFP signals were readily observed in CIK cells, a non-GSC control. When ricefield fGSCs were transfected, however, GFP signals were barely observed (Figure 2A). In the following days, few GFP-positive cells survived, and these cells lost their stem cell morphology, indicating spontaneous differentiation.

Next, electroporation transfection was performed using the Neon electroporator (Invitrogen™ Neon™ Transfection System, Thermo Fisher Scientific). Two days after transfection, the majority of CIK cells were GFP-positive. Although a transfection efficiency of approximately 70% was achieved, cell viability was less than 40%. Although approximately 40% of ricefield eel GSCs were GFP-positive after electroporation, more than 80% of the cells died within the next 2 days, indicating extensive cell damage caused by electroporation operation.

Then, microchannel-mediated cell transfection was performed. After 2 days, nearly 100% of CIK cells were GFP-positive. Importantly, the majority of fGSCs were also GFP-positive. These cells survived and proliferated well, displaying typical GSC morphology.

Taken together, the microchannel method achieved a notably high transfection efficiency (approximately 70%) in ricefield fGSCs, with high cell viability (75.9 ± 2.8%). We therefore chose to use the microchannel cell transfection system for the subsequent experiments in fish GSCs.

### 2.3. High Gene Editing Efficiency in Primary Monopterus albus Germ Stem Cells

**Figure A1:**
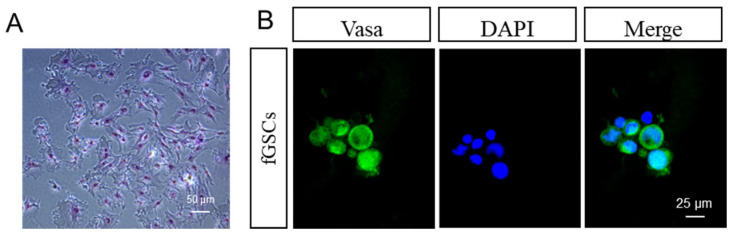
Alkaline phosphatase (AP) activity assay and Vasa immunofluorescence staining following microchannel transfection. (**A**) Representative AP staining images of fGSCs after microchannel transfection. (**B**) Representative Vasa Immunofluorescence staining images of fGSCs after microchannel transfection.

The pCas9-mU6sgRNA plasmids targeting *cyp19a1a* and *foxl2* were constructed and transfected into ricefield eel fGSCs using the microchannel cell transfection instrument. Two days after transfection, abundant green fluorescence was observed, with a transfection efficiency of approximately 70–90%. After 5–7 days of selection with 500 µg/mL G418, the transfected cells attached well to the plate, displaying typical stem cell morphology (Figure A1).

**Figure 3:**
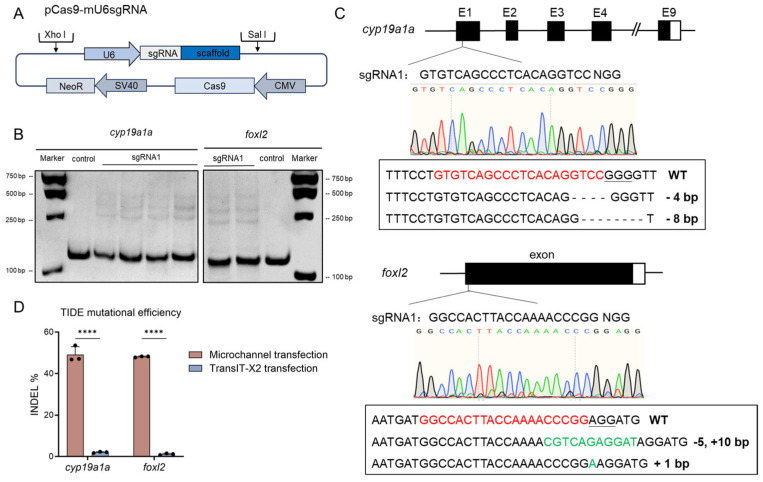
High gene editing efficiency in primary *Monopterus albus* germ stem cells. (**A**) The integrated pCas9-mU6sgRNA plasmids containing sgRNAs targeting different genes were constructed. This vector was constructed by Dr. Wei Jing’s team at Southwest University, in which Cas9 expression is driven by a CMV promoter, while sgRNA expression is under the control of a medaka-derived U6 promoter. (**B**) HMA (heteroduplex mobility assay). pCas9-mU6sgRNA plasmids targeting the endogenous genes *cyp19a1a* and *foxl2* were transfected into GSCs. After G418 selection for 5–7 days, genomic DNA was extracted, and the amplicons were separated by PAGE, giving a main band of homoduplex and slower-migrating bands corresponding to heteroduplex DNA. Control lanes show amplicons from wild-type GSCs. (**C**) Sanger sequencing of PCR products and single-colony sequencing. Green showing the exact nucleotides in 11- or 1 bp mutants that are different from the WT. Red showing the CRISPR targeting site (**D**) TIDE (Tracking of Indels by Decomposition) analysis of amplicons from GSCs transfected with the pCas9-mU6sgRNA vector after G418 selection. The TIDE analysis was performed on PCR amplicons derived from three independent biological replicates (*n* = 3 fish per group), and representative chromatograms are shown. The calculated indel mutation rates (mean ± SD) are indicated. ****: *p* < 0.0001.

Genomic DNA was extracted and subjected to HMA and TIDE analyses. The HMA results showed obvious retarded bands on the native polyacrylamide gel for the experimental groups, indicating the presence of heteroduplex DNA molecules (Figure 3B). The wild-type control group displayed a single, clear band. To confirm successful gene editing, Sanger sequencing of the PCR products was performed. Based on the sequencing chromatograms, the control group showed clear, single peaks in the target region, whereas the experimental group displayed overlapping peaks starting from the expected targeting sites (the third base upstream of the PAM), indicating the presence of various mutational alleles at the population level (Figure 3C). Furthermore, to confirm the mutations, PCR-amplified fragments near the target sites were purified, ligated into vectors, and sequenced (Figure 3C). The sequencing results revealed random insertions or deletions at both gene target sites, further confirming the effectiveness of this system. To assess the indel mutation frequency, the sequencing data were analyzed using TIDE software (version 5.0.2) (Figure 3D). The results showed that the indel mutation rates were 45–60%, depending on the target gene and conditions.

### 2.4. High Gene Editing Efficiency in Gibel Carp GSCs

To assess whether the microchannel cell transfection method is applicable to GSCs of other fish species, we repeated the experiments using medaka SG3 cells and GSCs from gibel carp (*Carassius gibelio*). In medaka, two sgRNAs targeting the *tmem104* and *ptch1* genes were used [13]. The corresponding pCas9-mU6sgRNA plasmids were constructed and transfected into SG3 cells using the microchannel cell transfection system.

**Figure 4:**
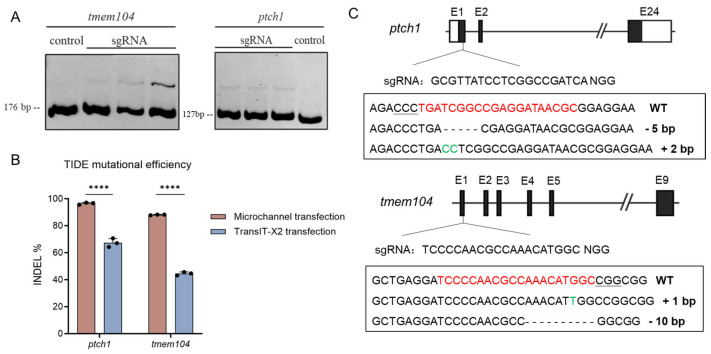
High gene editing efficiency in medaka SG3 cells. (**A**) HMA (heteroduplex mobility assay). pCas9-mU6sgRNA plasmids targeting the endogenous genes *tmem104* and *ptch1* were transfected into SG3 cells. After G418 selection for 7 days, genomic DNA was extracted, and the amplicons were separated by PAGE, giving a main band of homoduplex and slower-migrating bands corresponding to heteroduplex DNA. Control lanes show amplicons from wild-type SG3 cells. (**B**) TIDE (Tracking of Indels by Decomposition) analysis of amplicons from SG3 cells transfected with the pCas9-mU6sgRNA vector after G418 selection. Data are derived from three independent biological replicates (*n* = 3 independent transfection experiments). For each biological replicate, three technical replicates were performed. The mean indel frequencies are shown. (**C**) Mutation type detection of the endogenous genes *ptch1* and *tmem104*. ****: *p* < 0.0001.

**Figure A2:**
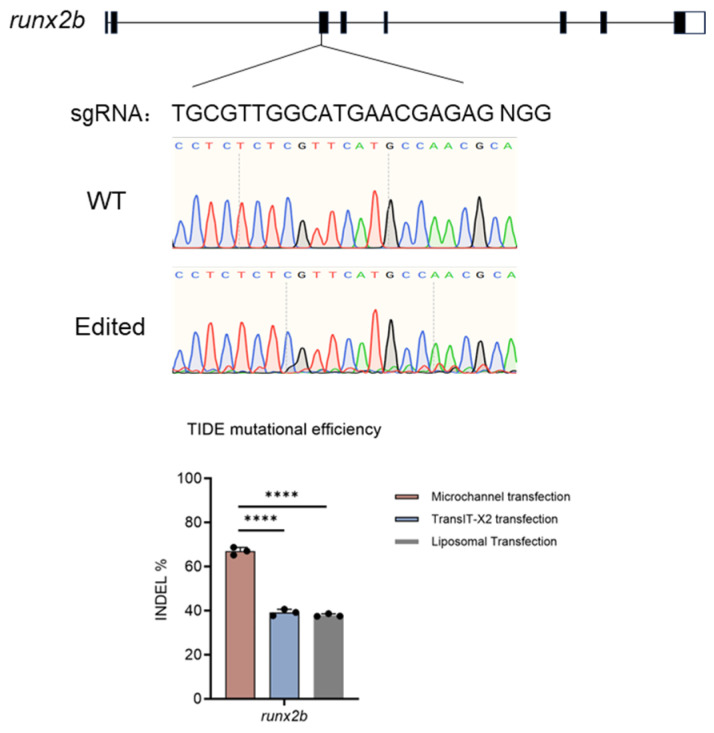
The Sanger sequencing of the PCR products and TIDE analysis data for gibel carp. ****: *p* < 0.0001.

Genomic DNA was extracted after selection with G418 for 5–7 days. DNA fragments around the target regions were amplified by PCR and subjected to HMA and TIDE analyses. The HMA results showed heteroduplex DNA formation in the experimental groups compared to the control. Sanger sequencing confirmed mutations in the genome of transfected SG3 cells. TIDE analysis revealed a mutational efficiency of 96.5% for the *ptch1* gene and 88% for the *tmem104* gene. Similar results were obtained using GSCs from gibel carp (Figure A2). The overall gene editing efficiency achieved with the microchannel cell transfection method was significantly higher than that achieved with chemical transfection reagents or electroporation (Figure 4).

## 3. Discussion

Previous work has shown that successful gene editing in fish cells is challenging. This is likely due to the difficulty in transfecting fish cells, and/or because the current Cas9 proteins and CRISPR plasmid vectors have not been optimized for fish cells. In this work, we report a systematic comparison of various gene delivery approaches in fish cultured GSCs and successfully establish an efficient, low-toxicity gene editing method using an integrated CRISPR/Cas9 vector strategy. Our results demonstrated that, compared to chemical reagents and electroporation, microchannel-mediated transfection led to much higher transfection efficiency with minimal cell death. This finding is consistent with reports on microfluidic transfection technology in mammalian cells [48], further validating the broad applicability of this technology in “hard-to-transfect” cell types. High gene editing efficiency can be achieved in multiple GSCs, including primary GSCs and the established SG3 line, demonstrating the general applicability of our method. Our results suggest that efficient transfection other than the current Cas9 proteins and CRISPR plasmid vectors is key to high gene editing efficiency in fish cultured cells. Importantly, after G418 selection, these cells survived, abundantly expressed Vasa, and showed stem cell-like characteristics, indicating that they remained in a stem cell state. These edited GSCs were expected to retain long-term germline competency after transplantation, which can be used to generate gene-modified gametes of ricefield eel.

Although CRISPR/Cas9 technology has been widely used in gene editing, its application in fish cultured cells remains relatively limited and faces numerous technical challenges. It involves several key steps, including cell transfection, screening of single-cell clones, and phenotypic identification of mutant cell lines. It is generally believed that transfection is key to high gene editing efficiency [49]. Compared to mammalian cells, fish cell membranes have higher phospholipid saturation, forming a tighter membrane structure that hinders the efficient delivery of foreign DNA. Simultaneously, fish cells are typically cultured at lower temperatures, further reducing membrane fluidity [12]. To overcome these barriers, researchers have attempted various strategies to deliver the CRISPR/Cas9 system into fish cells, such as the use of pre-assembled Cas9-sgRNA ribonucleoprotein complexes (Cas9-RNP) [1] and lentiviral transduction [3]. However, electroporation and chemical-mediated transfection methods often lead to high mortality and low efficiency, and viral vectors such as lentivirus raise biosafety concerns. Moreover, these methods often result in prolonged cellular stress, endosomal entrapment, and activation of differentiation pathways, which may compromise stem cell identity. The results of this study highlight the superiority of microchannel-based technology in stem cell transfection. The microchannel structure, by confining cell space and optimizing electric field distribution, enables efficient and uniform nucleic acid delivery at lower voltages, thereby maximizing transfection efficiency while minimizing cell damage. It also enables rapid and uniform delivery of nucleic acids with minimal mechanical and thermal stress, preserving the stem cell niche and reducing the risk of spontaneous differentiation. 

Despite the encouraging results, this study has limitations. First, we primarily used Vasa immunofluorescence and alkaline phosphatase (AP) staining to demonstrate the stemness of the GSCs before and after transfection. Antibodies for Nanos2 or Gfra1, which are “gold standard” GSC markers, Refs. [16,18] were not available for ricefield eel. Second, the indel mutation rate determined by TIDE varies, up to 60% in ricefield eel GSCs and up to 96.5% in the established SG3 cells. This variability suggests that the current gene editing system requires further optimization, especially in primary GSCs. Third, there is lack of in vivo transplantation validation. Although we have successfully established a GSC transplantation platform using intact GSCs from ricefield eel [47,50], GSC transplantation experiments were not performed to verify whether the gene-edited GSCs retain germline competency after transplantation, can differentiate into functional gametes, and produce heritable offspring. The transplantation of gene-edited GSCs is currently under investigation in our laboratory. 

In summary, this study provides an efficient and reliable technical solution for gene editing of fish GSCs. Future work should focus on in vivo functional validation, development of multi-gene editing strategies, and further optimization of the technology to facilitate its transition from laboratory research to industrial application.

## 4. Materials and Methods

### 4.1. Animal Ethics

All experimental procedures were strictly conducted in accordance with the guidelines of the Animal Care and Use Committee of the Institute of Hydrobiology, Chinese Academy of Sciences (Approval Code: IHB/LL/2022046), Wuhan, Hubei Province, China.

### 4.2. Cell Isolation and in Vitro Culture

Experimental ricefield eels, which were wild-caught, were purchased from the Baishazhou aquatic market (Wuhan, China). Germline stem cells from 3 healthy male (4-year-old, 50–55 cm) and 5 female (1.5-year-old, 15–20 cm) animals were isolated using a differential plating method [21] or a Percoll-mediated purification method as described below. Briefly, fish were anesthetized with MS222 (100 mg/L, Sigma, St. Louis, MO, USA), followed by dissection of gonads under a biological microscope (Mshot, Guangzhou, China). The gonadal cell suspension was obtained after enzymatic digestion of the gonads, followed by filtration through a 40 μm strainer. A total of 5 × 10^7^ gonadal cells were obtained, which likely included GSCs, blood cells, and gonadal somatic cells. The cell suspension was loaded onto a discontinuous Percoll density gradient (20%, 30%, 40%, and 60%; Sain Innovation, Beijing, China) and centrifuged at 800× *g* for 30 min. The resulting cell fractions were collected and transferred to a 15 mL centrifuge tube, followed by two washes with DPBS (Dulbecco’s Phosphate-Buffered Saline) for 2 min each. The cell pellet was transferred to a 6-well plate pre-coated with 0.1% gelatin and incubated at 28 °C for 2 days. The cells in the 40% Percoll fraction showed the highest expression levels of stem cell marker genes such as vasa. When cell confluence reached 90%, cells were treated with trypsin to dissociate into single cells and split at a 1:3 ratio. Ricefield eel GSCs were maintained in the modified GSC maintenance medium previously reported by our group [21]. The medium contained 85% RPMI 1640, 5% FBS, 5% KSR, 4% ricefield eel serum, 1% sodium pyruvate, 1% L-glutamine, 50 µM β-mercaptoethanol, 1% penicillin/streptomycin (P/S), 15 mM Hepes, 10 ng/mL LIF, 40 ng/mL FGF, and 40 ng/mL EGF. Cell morphology was monitored and recorded under a microscope.

### 4.3. Plasmids and sgRNA Design

The pCas9-mU6sgRNA plasmid for gene targeting was based on an integrated CRISPR/Cas9 vector, a gift from Prof. Jing Wei at Southwest University, China [13,49]. In this vector, Cas9 expression is driven by the CMV promoter, and sgRNA expression is driven by a medaka U6 promoter. The sgRNAs targeting the *cyp19a1a* and *foxl2* genes (Table A1) of *Monopterus albus* were synthesized and ligated into pCas9-mU6sgRNA using a seamless cloning kit (2×MultiF Seamless Assembly Mix, RK21020, Abclonal, Wuhan, China). For instance, to generate pCas9-mU6sgcyp19a1a, the pCas9-mU6sgRNA plasmid was linearized using the restriction enzymes Xho I and Sal I. The U6-sgRNA-scaffold was cut into two parts using Xho I and Sal I. To construct the U6-sgRNA-scaffold cassette for ricefield eel, two DNA fragments were amplified using the primers listed in Table 1. The upper fragment was amplified with the primer pair U6XhoI-F (5′-GAGTTCTTCTGAACGCGTCTCGAGCCTCTAGAACTATAGTGA-3′) and sgcyp19a1a-R1 (5′-AACCAACAGGAACTACTGCCTACGATGAGCCAAAGTCTCTGA-3′), while the lower fragment was amplified with the primer pair sgcyp19a1a-F1 (5′-TAGGCAGTAGTTCCTGTTGGTTGTTTTAGAGCTAGAAATAGC-3′) and scaffoldSal-R (5′-CGCCATATTGAATTGGCGGTCGACTGGCGTAATAGCCAACCTT-3′). The three DNA fragments were assembled according to the manufacturer’s instructions (ABclonal 2×MultiF Seamless Assembly Mix, RK21020) and sequenced. All gRNA sequences are listed in Table 2.

The pCS2-eGFP plasmid (No. 221710) was purchased from Addgene (www.addgene.org) and used to monitor transfection efficiency.

### 4.4. Cell Transfection Using Different Methods

Three different cell transfection methods (chemical transfection, electroporation, and microchannel-based transfection) were applied to fish cultured cells. One day before transfection, approximately 1 × 10^6^ ricefield eel GSCs, CIK cells, and SG3 cells were plated in a 6-well plate. CIK (*Ctenopharyngodon idellus* kidney) cells, derived from grass carp kidney, were used as a non-GSC control. The medaka (*Oryzias latipes*) spermatogonial stem cell line (SG3) was used as a control for established GSCs. The next day, transfection was performed when cells reached 80–90% confluence. The pCS2-eGFP plasmid was transfected into the target cells to monitor transfection efficiency using the methods described below.

#### 4.4.1. The Chemical Transfection Group

We used at least five different chemical transfection reagents, including Lipofectamine 3000 from Thermo Fisher Scientific, Waltham, MA, USA. Similar results were obtained with each reagent. Here we show the detailed protocol for three reagents.

TransIntro EL Transfection Reagent (TransGen Biotech, FT201, Beijing, China). According to the manufacturer’s instructions, plasmid DNA (4 µg) was diluted in 50 µL of Opti-MEM medium and gently mixed. Then, 8 µL of TransIntro EL was added to the diluted DNA and gently mixed. The mixture was incubated at room temperature for approximately 15 min and added dropwise to the cells with gentle shaking. The cells were cultured at 28 °C in a 5% CO_2_ incubator (Healforce, HF240, Shanghai, China) for 48 h before examination under a fluorescence microscope.

Hieff Trans™ Liposomal 2000 Transfection Reagent (YEASEN, 40802ES03, Shanghai, China). According to the manufacturer’s instructions, 3 µg of plasmid DNA was diluted in 250 µL of Opti-MEM serum-free medium and gently mixed. At the same time, 7.5 µL of Hieff Trans™ Liposomal Transfection Reagent was diluted in 250 µL of Opti-MEM serum-free medium, gently mixed, and kept for 5 min at room temperature. The DNA and diluted liposomal reagent were combined, gently mixed, and incubated at room temperature for 20 min. The resulting complex was added to the cells with gentle shaking. The cells were cultured at 28 °C in a 5% CO_2_ incubator for 48 h before examination under a fluorescence microscope.

TransIT-X2 Dynamic Delivery System (Mirus Bio, Madison, WI, USA). According to the manufacturer’s protocol, the TransIT-X2 reagent was warmed to room temperature and gently vortexed before use. Then, 250 µL of Opti-MEM serum-free medium was placed in a sterile tube, and 2.5 µg of plasmid DNA was added, followed by gentle pipetting to mix thoroughly. Then, 7.5 µL of TransIT-X2 was added to the diluted DNA mixture and gently pipetted to mix. After incubation at room temperature for 15–30 min, the complex was added dropwise to the cultured cells and mixed evenly. The cells were incubated at 28 °C in a 5% CO_2_ incubator for 48 h.

#### 4.4.2. The Electroporation Group

The electroporation system (Invitrogen Neon™ Transfection system, Thermo Fisher Scientific, Waltham, MA, USA) was used for electroporation. For ricefield eel GSCs, we tried different parameters (voltage: 1000–1600 V, pulse number: 1–4, pulse width: 5–30 ms), and similar results were obtained. The parameters in most experiments are shown in the Table 3 below:

After electroporation, following the manufacturer’s instructions, the cells were incubated at 28 °C in a 5% CO_2_ incubator for 48 h before examination under a fluorescent microscope.

#### 4.4.3. The Microchannel Cell Transfection Group

Transfection was performed using a microchannel cell transfection instrument (MDMP E-1, Mengde Biotechnology, Shanghai, China), following the manufacturer’s instructions. Briefly, one day before transfection, approximately 1 × 10^6^ ricefield eel GSCs were plated in a sample chamber with microchannel substrate. Transfection was conducted when cell confluence reached 80–90%. Before transfection, the complete medium was replaced with serum-free Opti-MEM. The instrument parameters were set as follows: LEVEL = 3, CYCLE = 2. The sample chamber was placed into the instrument, and transfection was performed. The medium was then replaced with complete medium (without 2% penicillin-streptomycin) and returned to the CO_2_ incubator for 48 h before examination under a fluorescent microscope. The cells were further cultured for 5–7 days in GSC maintaining medium containing 500 µg/mL G418.

Cell viability was assessed using a Trypan Blue exclusion assay 48 h post-transfection. Both live and dead cells were counted using a hemocytometer, and the survival ratio was calculated as: Viability (%) = (number of live cells/total number of cells) × 100. Three independent biological replicates (cells isolated from three individual fish) were performed, with three technical replicates per biological replicate. The percentage of EGFP^+^ cells was determined by counting at least 500 cells from five random fields per well under a fluorescence microscope.

### 4.5. Alkaline Phosphatase (AP) Staining

The alkaline phosphatase activity assay was performed using a cellular alkaline phosphatase kit (Mak530, Sigma, USA) according to the manufacturer’s instructions.

### 4.6. Immunofluorescence (IF) Staining

The cultured GSCs were fixed with 4% paraformaldehyde (PFA). After washing three times for 5 min each with PBST (Phosphate-Buffered Saline with 0.1% Triton X-100), the cells were blocked using 5% normal horse serum in PBST. Subsequently, the cells were incubated with a primary antibody against Vasa (DIA-AN, 3008, 1:200, Wuhan, China) overnight at 4 °C. Following three PBST washes, the cells were incubated with a secondary antibody (Alexa Fluor 488, Life Technologies, 1:500 in blocking buffer) for 1 h at room temperature in the dark. Nuclei were counterstained with DAPI (Sigma, St. Louis, MO, USA, 1:1000). Images were captured using a laser scanning confocal microscope (Leica, Wetzlar, Germany).

### 4.7. The Heteroduplex Mobility Assay (HMA)

Several mutation detection methods have been developed for the screening of CRISPR/Cas9-induced mutants, including heteroduplex mobility assay (HMA) on polyacrylamide gel electrophoresis. We employed the HMA to detect the mutation induced by the CRISPR/Cas9 system. Briefly, genomic DNA was extracted from the transfected cells. PCR with a 2×Taq Premix (Vazyme, Nanjing, China) was performed for 35 cycles (95 °C for 15 s, 60 °C for 15 s, 72 °C for 30 s) using the gene-specific primer sets (Table 2). Subsequently, 5 µL of the DNA fragments was loaded and separated by 15% polyacrylamide gel electrophoresis (PAGE) in TBE buffer using a PowerPac basic electrophoresis apparatus (Bio-Rad, Hercules, CA, USA). Images were captured and recorded using a gel imaging system (Peiqing, JS-012, Shanghai, China).

### 4.8. The TIDE Analysis

TIDE (Tracking of Indels by Decomposition) was used to assess the insertion/deletion (indel) mutation frequency (http://tide.nki.nl, version 5.0.2), 5 June 2025. The PCR amplicons of DNA from control and gene-edited cells were purified (Vazyme, Nanjing, China) and sent to Tsingke Biotechnology Co., Ltd. (Beijing, China) for Sanger sequencing. The resulting chromatogram files were uploaded to the TIDE website and analyzed using the default settings, except that the INDEL size range was set to ±50 bp.

### 4.9. Statistical Analysis

All data were processed using GraphPad Prism software (version 9.5.1). Data are presented as mean ± standard deviation (SD). Three independent biological replicates were performed for all experiments unless otherwise specified. For each biological replicate, three technical replicates were performed. Statistical analysis was performed using Student’s *t*-test or one-way ANOVA followed by Tukey’s post hoc test, with the criterion for statistical significance set at *p* < 0.05.

## 5. Conclusions

We show that microchannel-based technology is superior to chemical reagents and electroporation in mediating CRISPR/Cas9 vector-based gene editing in *Monopterus albus* germline stem cells. It enables (1) high transfection efficiency with a low cell death rate, (2) highly efficient gene editing within one week, and (3) effective maintenance of the stemness and proliferative potential of the gene-edited germ stem cells. This study established an efficient and reliable gene editing system for fish GSCs, which will be useful for speeding up the creation of new germplasm of genetically difficult-to-breed fish by combining GSC transplantation with gene editing techniques.

## Figures and Tables

**Table 1:** Information of primers in this work.

Primer	Primer Sequence
U6Xho1-F	GAGTTCTTCTGAACGCGTCTCGAGCCTCTAGAACTATAGTGA
scaffoldSal-R	CGCCATATTGAATTGGCGGTCGACTGGCGTAATAGCCAACCTT
sg*cyp19a1a*-R1	AACCAACAGGAACTACTGCCCGATGAGCCAAAGTCTCTGAG
sg*cyp19a1a*-R2	GGTCTGCCGCTGCTGCATCCCGATGAGCCAAAGTCTCTGAG
sg*foxl2*-R1	AAACAAAACAAGTTCAGTCCCGATGAGCCAAAGTCTCTGAG
sg*foxl2*-R2	CCGGGTTTTGGTAAGTGGCCCGATGAGCCAAAGTCTCTGAG
sg*ptch1*-R	TGATCGGCCGAGGATAACGCCGATGAGCCAAAGTCTCTGA
sg*tmem104*-R	GCCATGTTTGGCGTTGGGGACGATGAGCCAAAGTCTCTGA
sg*cyp19a1a*-F1	GGCAGTAGTTCCTGTTGGTTGTTTTAGAGCTAGAAATAGC
sg*cyp19a1a*-F2	GGATGCAGCAGCGGCAGACCGTTTTAGAGCTAGAAATAGC
sg*foxl2*-F1	GGACTGAACTTGTTTTGTTTGTTTTAGAGCTAGAAATAGC
sg*foxl2*-F2	GGCCACTTACCAAAACCCGGGTTTTAGAGCTAGAAATAGC
sg*ptch1*-F	GCGTTATCCTCGGCCGATCAGTTTTAGAGCTAGAAATAGC
sg*tmem104*-F	TCCCCAACGCCAAACATGGCGTTTTAGAGCTAGAAATAGC
*cyp19a1a*-PAGE-F	GGCAAGTTGCTTTCAGTCTGC
*cyp19a1a*-PAGE-R	CTCTGGTTGCCACTGAGATG
*foxl2*-PAGE-F	GCGCCTTTAACACTTCTGCTT
*foxl2*-PAGE-R	CTTTGGGTCGCTCTTTCTCCT
*ptch1*-PAGE-F	CTAATGCACCCTCCGAACAG
*ptch1*-PAGE-R	ATCGCAGTAACTCGGTCGCT
*tmem104*-PAGE-F	AACCTTCTCTCACACGGCAG
*tmem104*-PAGE-R	TCACAAACGGAGAGTACGGC
*cyp19a1a*-TIDE-F	CCGACTGCTGAGGCAACTTT
*cyp19a1a*-TIDE-R	CCATTGACAGGTACACCAAGG
*foxl2*-TIDE-F	CGGTGCATCCTGAAGTTCTAC
*foxl2*-TIDE-R	CGCTCCGTTACTGGAGGAGA
*ptch1*-TIDE-F	AGCCGAAGACTCGTGTATGG
*ptch1*-TIDE-R	CAGGTTAACGCAGAAGCCAC
*tmem104*g1-TIDE-F	GTGTGGTTACATGCGAAGGTG
*tmem104*g1-TIDE-R	CTGACGAGTGCAGAAGGTGA

**Table 2:** Target site sequences in the present study.

Gene		Target Sequence
*cyp19a1a*	Target a	GGCAGTAGTTCCTGTTGGTT NGG
	Target b	GGATGCAGCAGCGGCAGACC NGG
*foxl2*	Target a	GGACTGAACTTGTTTTGTTT NGG
	Target b	GGCCACTTACCAAAACCCGG NGG
*trpv4*	Target a	AGGAGCGCTCACCAGGCGAA NGG
	Target b	GATTTGGGGGTGGGACAGAG NGG
*tmem104*	Target a	TCCCCAACGCCAAACATGGC NGG
*ptch1*	Target a	GCGTTATCCTCGGCCGATCA NGG
*runx2b*	Target a	TCAGAGAAGAGTCCGGCCTT NGG

**Table 3:** The parameters for electroporation.

Pulse Voltage (V)	Pulse Width (ms)	Pulse Number	Cell Density (cells/mL)	Plasmid DNA	Tip Type
1400	10	3	1 × 10^7^	0.5 µg	10 µL
1200	20	2	1 × 10^7^	0.5 µg	10 µL

## Data Availability

Raw Sanger trace files and source data are available from the corresponding author upon reasonable request. All other data generated or analyzed during this study are included in the published article.

## Abbreviations

The following abbreviations are used in this manuscript:

WT	Wild type
CRISPR	Clustered Regularly Interspersed Short Palindromic Repeats
Cas9	CRISPR-associated 9
PCR	Polymerase Chain Reaction
gRNA	guide RNA
IF	Immunofluorescence
DPBS	Dulbecco’s Phosphate Buffered Saline
FBS	Fetal Bovine Serum
GSCs	Germline Stem Cells
SSCs	Spermatogonial Stem Cells
FGSCs	Female Germline Stem Cells
HMA	Heteroduplex Mobility Assay
TIDE	Tracking of Indels by Decomposition
PAGE	polyacrylamide gel electrophoresis
Amp	Ampicillin
DAPI	4′,6-diamidino-2-phenylindole
AP	Alkaline phosphatase
RNP	ribonucleoprotein complex

## Appendix A

**Table A1:** Gene information for *cyp19a1a* and *foxl2* in the ricefield eel (*Monopterus albus*).

Gene	Gene Description	Gene Type	Location	Exon Count	Paralogs
*cyp19a1a*	cytochrome P450, family 19, subfamily A, polypeptide 1a	protein coding	NW_018127952.1 (378551..381443, complement)	9	*cyp19a1b*
*foxl2*	forkhead box L2a	protein coding	NW_018127888.1 (1974161..1976200, complement)	1	*foxl2b*
